# A hybrid deep learning approach with temporal awareness for intelligent intrusion detection in 6G-enabled IIoT networks

**DOI:** 10.1038/s41598-026-43058-x

**Published:** 2026-03-14

**Authors:** Gaoyang Guo, Faizan Qamar, Syed Hussain Ali Kazmi, Fazlina Mohd Ali, Ihsan Ali

**Affiliations:** 1 Center for Cyber Security, Faculty of Information Science and Technology, Universiti Kebangsaan Malaysia (UKM), UKM Bangi, 43600 Selangor Malaysia; 2 Center for Software Technology and Management, Faculty of Information Science and Technology, Universiti Kebangsaan Malaysia (UKM), UKM Bangi, 43600 Selangor Malaysia; 3 Department of Computer Science, Southeast Missouri State University, Cape Girardeau, 63701 MO USA

**Keywords:** 6G, Industrial IoT, Deep learning, Intrusion detection, Neural networks, Hybrid model, Engineering, Mathematics and computing

## Abstract

The integration of the sixth-generation (6G) communication technology and the Industrial Internet of Things (IIoT) has realized the intelligence and automation of industrial applications. However, due to the complexity, dynamics, and heterogeneity of data, traditional threat detection methods make it difficult to deal with cyber threats in the 6G-IIoT environment. In view of these limitations, this study proposes a hybrid Deep Learning (DL) model combining a Deep Neural Network (DNN), a Bidirectional Gated Recurrent Unit (BiGRU), and an attention mechanism for threat detection in a 6G-IIoT environment. DNN extracts global features, BiGRU captures bidirectional temporal dependencies, and the attention mechanism highlights key anomalies. Experimental results on the Edge-IIoTset dataset show that the accuracy rate of the model is $96.88\%$. It outperforms baseline models (such as ANN, CNN, DNN-LSTM). The model achieves high accuracy and low False Positive Rate (FPR), and meets the dynamic security requirements of the 6G-IIoT environment. This research provides a promising solution for real-time threat detection in next-generation industrial networks.

## Introduction

### Background

With the continuous development of Industry 4.0 and intelligent manufacturing, the Industrial Internet of Things (IIoT) is developing at a high speed^1^. Various sensors and devices have been widely used in industrial production, which promotes the rapid development of emerging application scenarios such as intelligent manufacturing, autonomous driving, and telemedicine^2^, and realizes real-time data acquisition, analysis, and intelligent processing. The fifth-generation (5G) communication technology is transitioning to the sixth-generation (6G) communication technology^3^, which will realize Enhanced Ultra-Reliable Low-Latency Communication (eURLLC), Ultra-Massive Machine-Type Communication (umMTC), and Future Enhanced Mobile Broadband (FeMBB) in 6G^4^. The development of these technologies is revolutionizing the application of IIoT, enabling industrial systems to reach a higher level of automation, collaboration, and intelligent decision-making.

However, although the deep integration of 6G and IIoT improves the intelligence level of industrial systems, it also brings new network security challenges^5^. Firstly, the complexity, dynamics, and heterogeneity of data in the 6G-IIoT network environment have increased significantly, and traditional threat detection methods have made it difficult to comprehensively capture and analyze the potential attack behavior in this multi-dimensional data^6^. Secondly, the diversity of communication protocols and equipment in industrial networks also makes conventional security protection methods have limitations, which cannot adapt to the network security requirements in large-scale and complex scenarios in the future^7^.

Deep learning (DL) is a solution to solve the complex security requirements of the next-generation network. By using the power of neural networks, large amounts of data can be analyzed to identify and detect anomalies, which shows significant advantages in the field of intrusion detection^8^. However, a single model still has limitations when dealing with high-dimensional heterogeneous data in 6G-IIoT scenarios^9^. Researchers try to improve the detection performance by combining hybrid models with multi-branch architectures^10–13^. In addition, the 6G-IIoT environment has strict requirements for real-time performance and False Positive Rate (FPR) control, which further increases the complexity of model design.

### Motivation and contribution

The rapid development of 6G has promoted the development of IIoT. However, the complexity of the 6G-IIoT network environment makes it extremely vulnerable to complex network attacks, and traditional threat detection methods are not enough to address dynamically evolving and complex network threats. Therefore, the 6G-IIoT environment requires an advanced threat detection technology that can adapt to emerging cyber threats. DL is a promising solution due to its ability to efficiently analyze large-scale data and detect anomalous behavior. However, existing DL models have limitations for threat detection in 6G-IIoT networks, which motivates the exploration and development of new DL models. The key contributions of this work are as follows:

- We propose a novel hybrid DL architecture, the DNN-BiGRU-Attention (Deep Neural Network and Bidirectional Gated Recurrent Unit with Attention) model, which can effectively identify complex and unknown threats in 6G-IIoT networks.
- We evaluate the proposed model by comparing it with baseline models using a public IIoT dataset, and the results confirm its effectiveness in enhancing network security.
- We analyze the model’s practicality in 6G-IIoT security and point out potential future research directions, all of which are promoting the practical application of DL in the next-generation industrial network security.

### Organization and structure

This paper has five sections, and the structure details are as follows. The first section is an introduction. Related work is presented in Section “Related work”. The methodology is presented in Section “Methodology”, including the dataset description and preprocessing, the proposed model, experimental design, and the evaluation metrics. In Section "Results and discussion", the evaluation results are presented, and the proposed model was compared with the baseline and the state-of-the-art study, and then the results are discussed. Section "Conclusion and future work" concludes the paper with conclusions and future work.

## Related work

Positioned as the successor to 5G technology, 6G networks aim to deliver enhanced wireless communication capabilities through four key advancements: ultra multi-gigabit data speeds, sub-millisecond latency, massive device connectivity, and ubiquitous network coverage^14^. 6G innovation is also fueled by converged technologies, including blockchain, Artificial Intelligence (AI), and digital twins^15^, enabling the adoption of the IIoT^16^.

### Security challenges in 6G industrial IoT

IIoT, as an essential part of the digital transformation in the industrial field, relies on the interconnectivity and real-time data analysis of smart devices and has been widely applied in manufacturing, agriculture, transportation, and other fields^17,18^. However, this highly complex and interconnected industrial ecosystem has also led to new security challenges, including distributed denial of service (DDoS) attacks, data privacy and leakage, injection attacks, man-in-the-middle (MITM) attacks, and malware^19^. Furthermore, the progress of 6G networks has made the IIoT environment more vulnerable to new attack vectors, ranging from malware attacks to Advanced Persistent Threats (APT)^20^. As a core technology in 5G and 6G systems, network slicing introduces additional security complexities, including slice isolation issues, vulnerabilities in Software Defined Networking (SDN) and Network Functions Virtualization (NFV), and multi-tenancy challenges, expanding the attack surface^21^. Due to the scale and complexity of threats in 6G-IIoT, a stronger, adaptive, and automated detection mechanism is required, which calls for new technologies to adapt to the ever-changing network threats.

### Traditional methods and limitations

Traditional threat detection methods in IIoT networks mainly rely on rule-based or signature-based methods, which identify potential threats by matching the definitions or characteristics of known threats. Although these traditional techniques are effective against known threats, detecting unknown attack vectors and changing threats is difficult due to their reliance on rule sets and signature libraries^22^. So, applying traditional methods to the 6G-IIoT network environment is a major challenge, and a major drawback is the inability to adapt to the unknown network threats^23^. Another key issue is that processing the massive amount of real-time data generated by IIoT devices may be difficult, leading to performance bottlenecks and delayed threat response.

Moreover, when new threats emerge, traditional methods usually require frequent updates to keep rules and signatures valid, increasing maintenance overhead^24^. Traditional methods are also poor in detecting complex attacks such as zero-day attacks and APT^25^. Recent research has shown that signature-based Intrusion Detection Systems (IDS), such as Snort, have an accuracy of only $17\%$ on zero-day attacks, while DL-based methods can detect such unknown cyber threats with significantly improved accuracy^26^. Other studies have also demonstrated the effectiveness of DL methods in detecting zero-day attacks. For example, in study^27^, Almuflih et al. combined an attention-based bidirectional GRU with feature selection and hyperparameter optimization techniques to propose a Binary Snake Optimizer with DL-Enabled Zero-Day Attack Detection and Classification (BSODL-ZDADC) method. This method can effectively detect unknown attack behaviors (including zero-day attacks). However, the limitations of these traditional methods highlight the necessity of DL approaches that can learn patterns from data and adapt to changing threats without manually updating the rules.

### Deep learning for IIoT security

In the related research on applying deep learning in IIoT security, scholars have proposed various improved models to address the challenges of cyber threats, which are summarized in Table 1. For example, Oyinloye et al.^28^ improved the anti-interference ability and computational efficiency of the IDS by introducing random weights and standardized scalers through the improved ANN model and achieved an accuracy rate of $92\%$. However, the interpretability of their model is insufficient, and they still face the risk of adversarial attacks. The research by Shnain et al.^29^ combined Edge Computing (EC) with the improved Faster Recurrent Convolutional Neural Networks (Faster R-CNN) model, used CNN to extract hierarchical features, and processed IIoT traffic data through the edge server, and achieved $93.77\%$ accuracy and $91.03\%$ F1-score in malware detection, which significantly improved the detection speed. However, the model has a high demand for computing resources. In a study, Li et al.^30^ designed a bidirectional long and short-term memory network with multi-feature layers based on multi-feature layers (B-MLSTM). By introducing sequence and stage feature layers and double backward units, the learning ability and generalization ability of the model were improved. This design improves the detection accuracy of attacks at different time intervals, significantly reduces the FPR and False Negative Rate (FNR), and the accuracy reached $95.01\%$. However, the complex structure of the B-MLSTM model leads to a long training time and high computational cost.

In addition, the Snapshot Ensemble Deep Neural Network (SEDNN) model designed by Rouzbahani et al. in study^31^ realizes the detection of network attacks with $90.58\%$ accuracy and $90.48\%$ F1-score by integrating multiple local optimal models, and reduces the training time, but its generalization ability across datasets needs to be verified. The PIGNUS model proposed by Jayalaxmi et al. in study^32^ combines Autoencoder (AE) and Cascaded Forward-Back Propagation Neural Networks (CFBPNN), which are optimized in feature selection and attack detection stages, respectively, and achieve more than $95\%$ accuracy and $20\%$ FPR reduction, but it relies on high-quality training data and requires high computing resources.

The recent research indicates that hybrid DL models are gradually becoming the mainstream approach for threat detection in the IIoT. For example, Gulzar et al. in the research^33^ proposed a hybrid learning model named DeepCLG, which integrates CNN, LSTM, GRU, and Capsule Network (CN) and uses Boruta feature selection technology to extract significant features and shorten the training time automatically. This model achieved a high accuracy rate of $99.82\%$ on the CICIoT 2023 dataset, but it has the drawbacks of complex parameter selection and high computational complexity. In the other research^34^, Anwar et al. designed a Federated Learning (FL) with a CNN-Bidirectional LSTM (CNN-Bi-LSTM) model, which combines the advantages of CNN and Bi-LSTM and can identify complex attacks in IIoT networks. It significantly enhances data privacy protection through FL technology and reduces communication overhead. The model achieved an accuracy rate of up to $97.8\%$ on the X-IIoTID dataset. However, this model has high cumulative communication overhead during repeated synchronous large model updates and high computational resource requirements for the model.

These studies show that existing methods have made progress in improving detection performance, but are still limited by problems such as model interpretability, resource consumption, and data quality dependence. More efficient and robust hybrid architectures are urgently needed to deal with dynamic threats in IIoT environments.

**Table 1:** Summary of the deep learning for IIoT security.

Author	Model	Advantages	Limitations	Performance metrics
Oyinloye, T. S. et al^28^.	ANN	Improve anti-interference ability and computational efficiency.	The model is not interpretable enough.	Accuracy: 92%.
Shnain, A. H. et al^29^.	Faster R-CNN	Extract hierarchical features, and accelerate malware detection.	High demand for computing resources.	Accuracy: 93.77%, F1-score: 91.03%.
Li, X. et al^30^.	B-MLSTM	The sequence and stage feature layers and double backward units were introduced to enhance the learning ability and generalization ability.	The complex structure of the model leads to long training time and high computational cost.	Accuracy: 95.01%.
Rouzbahani, H. M. et al^31^.	SEDNN	Shorten training time.	The cross-dataset generalization ability needs to be verified.	Accuracy: 90.58%, F1-score: 90.48%.
Jayalaxmi et al^32^.	PIGNUS (AE+CFBPNN)	Feature selection and attack detection phase optimization to reduce false positive rate.	It relies on high-quality training data and has high computational resource requirements.	Accuracy: 95%.
Gulzar et al^33^.	DeepCLG (CNN+LSTM+GRU)	Automatic feature extraction; reduced training time.	Complex parameter selection and high computational complexity.	Accuracy: 99.82%.
Anwar et al^34^.	CNN-Bi-LSTM	Identifying complex attacks in IIoT networks; Enhancing data privacy protection; Reducing communication costs.	There are issues of high cumulative communication costs and high computational resources required.	Accuracy: 97.8%.

## Methodology

**Fig. 1:**
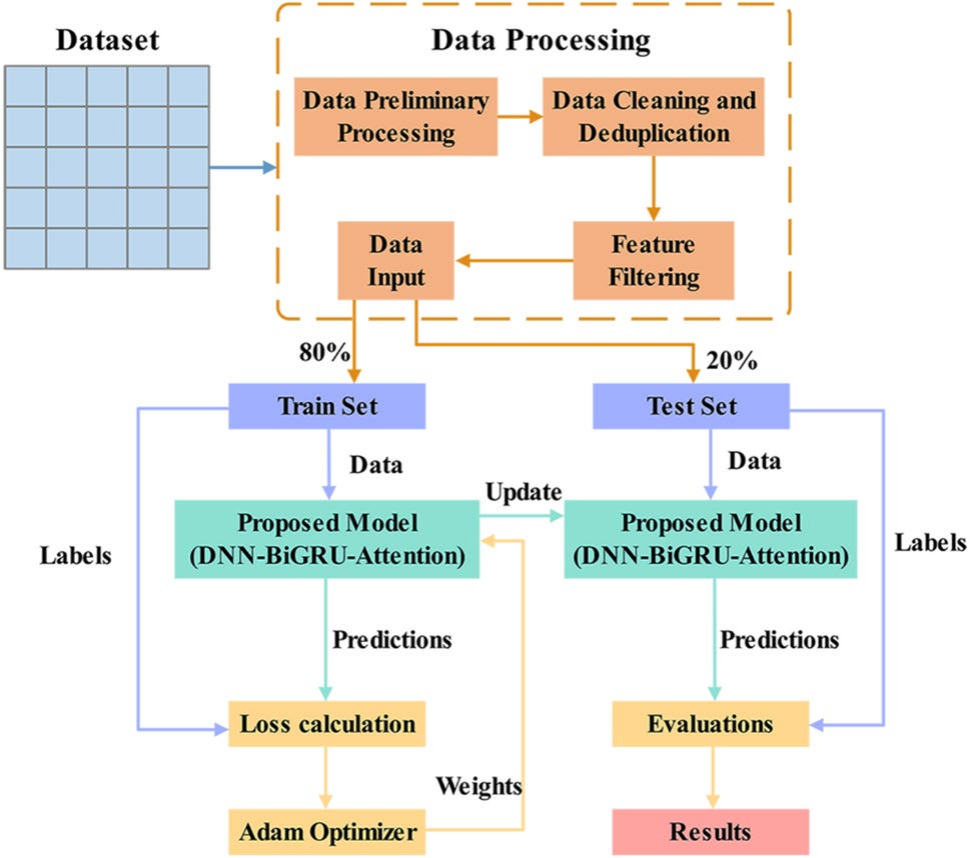
The proposed architecture flowchart.

The key stages of this section are shown in Fig. 1. First, the dataset used in this study was analyzed and preprocessed. Second, training and testing sets were created from the preprocessed dataset. Finally, the proposed model undergoes training and testing processes iteratively until the final results are output.

### Dataset description

In this study, data preprocessing is the key stage to ensure the model performance and the evaluation reliability. This experiment utilizes the Edge-IIoTset dataset proposed by M. A. Ferrag et al.^35^, which is a cybersecurity dataset that can be used in both ML and DL models. The dataset contains various attack types, including DDoS, man-in-the-middle attacks, injection attacks, information gathering, and malware attacks, and multiple features. However, this dataset contains far more samples of normal traffic than attack traffic, making it a highly imbalanced dataset. There is a significant risk that using simple random sampling to split the data, because of the single randomness, some rare classes of attacks may not be assigned to the training or test set at all, resulting in severely biased and unreliable model evaluation results.

To address this problem, this study used a stratified sampling strategy to divide the dataset into $80\%$ training set and $20\%$ test set. Specifically, stratified sampling is a data selection technique that divides the dataset into distinct, homogeneous subgroups, called strata, and then samples proportionally or optimally from each subgroup^36^. In other words, stratified sampling does not involve uniformly and randomly selecting samples from the entire dataset, stratified sampling first organizes the data into clusters or groups with similar characteristics^37^. In DL, this approach is mainly used to improve training efficiency, reduce variance in gradient computation, and handle imbalanced datasets^37,38^. It also ensures that the resulting samples are more representative of the overall structure than simple random sampling.

We didn’t use K-Fold Cross-Validation (K-Fold CV), which is the gold standard for evaluating generalization ability. Because standard K-Fold CV randomly shuffles the data and divides it into K-Folds, this breaks the inherent temporal order of the data^39,40^. This causes the model to see “future” data during training time and predict “past” data during testing, resulting in inflated and unreliable evaluation results. Secondly, Edge-IIoTset is a large-scale dataset containing millions of instances, while our proposed hybrid DL model training process is computationally intensive and uses K-Fold cross-validation, which results in a K-Fold increase in the total training time, which is infeasible in resource-limited IIoT environments.

### Dataset preprocessing

A key process in training and testing a DL model is dataset preprocessing. If the dataset is not handled correctly, the DL model will not function effectively. Based on the format of the dataset, the data preprocessing is divided into seven steps:

1. Data Import and Preliminary Processing: The original dataset is imported into memory. Due to the large size of the dataset, considering that there may be a large amount of data and mixed types in the dataset. ’low_memory=False’ is a common parameter in pandas (an extension library for Python used in data analysis) that aims to sacrifice memory (use more memory) for data type accuracy and avoiding warnings^41^. It ensures all data is loaded correctly and consistently, preventing subsequent code from encountering errors due to mixed data types. Specifically, when pandas reads large CSV files, it reads and parses data in chunks to save memory. Since the CSV file itself does not have a strict data type definition, the ’low_memory=False’ argument forces pandas to read the entire file into memory at once before parsing the data type^42^. Then, by looking at all the rows, pandas can make a final, accurate data type determination for each column. If we leave the default value, which is ’low_memory=True’, pandas will read the data in a more memory-efficient way, resulting in a chunk-inferred type, raising a DtypeWarning.
2. Column Name Renaming: Some column names in the original dataset are prone to confusion; in order to enhance the readability of data processing and facilitate subsequent operations, the column names in the dataset were renamed for some of the data. Specifically, we renamed some columns with verbose original names or containing special characters (such as dots). For example, “http.request. method” was renamed to “http1”, and “dns.qry.name.len” was renamed to “dns”, thereby improving the readability of the code and the convenience of subsequent operations.
3. Category Variable Encoding: Label encoding and one-hot encoding were used to transform the categorical features into data suitable for DL processing. If the output of label encoding is directly used as the input to the model, the model may incorrectly learn an artificial sequential relationship. Although one-hot encoding increases dimensionality, it is the mathematically correct way to handle nominal categorical data^43^. Specifically, it transforms each class into a vector that is equidistant (or orthogonal) to all other classes, thus ensuring that the model treats each class as independent and equal, avoiding bias introduced by incorrect order relationships. Specific steps are as follows: First, label encoding was used to map each category to a unique whole number, and an encoder was created for each type of variable; the encoded results were added to a new column. Then, to avoid the order relationship between categories affecting the model, the integer encoding was converted into a unique encoding.
4. Data Cleaning and Deduplication: Missing values can be a form of information in themselves, and using imputation can create “spurious” data. This spoof data might mask genuine attack features or introduce new noise. Given the large size of our dataset, deleting rows containing missing values only results in a small amount of data loss, which will not significantly affect the model’s overall learning ability. Therefore, in dataset preprocessing, it is very important to ensure the uniqueness and integrity of the data. First, check each dataset column for any missing values. Rows containing any missing values were removed to ensure data quality for model training. Then, the number of duplicate records in the dataset is counted, and all duplicate records are removed to ensure the uniqueness of the data in the dataset.
5. Identification and deletion of the same column: To reduce feature redundancy and improve the training efficiency of the model, a method based on hash values was used to identify columns with identical content in the data frame. Column groups with identical content were identified by traversing all columns and comparing their hash values. By retaining only the first column of each group and deleting the remaining columns. The dimensionality of the feature space is reduced by keeping only the first column and removing the remaining columns. Specifically, first, we define a function to calculate the MD5 hash of all contents of a complete column. Second, we iterate through all columns, calculate their hashes, and use a dictionary to map the hashes to a list of column names. Then, we iterate over the groups, and if a hash value corresponds to multiple column names, it means the contents of these columns are exactly the same, because the hash value is unique and is the “fingerprint” of the data. Therefore, we only keep the first column of each group as a representative and delete all other columns in that group.
6. Removal of irrelevant features: Based on the analysis results of the dataset, further irrelevant or less influential features were removed from the model prediction. High-Cardinality identifiers, such as time stamp information (e.g., frame.time), source and destination host information (e.g., ip.src_host, ip.dst_host), have almost as many unique values as there are rows, and if they were introduced into the model, it would lead to a “curse of dimensionality” and severe overfitting. protocol-specific payload information (e.g., tcp.payload, mqtt.msg), these columns contain raw, unstructured data, so we chose not to use these raw payload columns.
7. Shuffle the data: To avoid the potential order of the data in the preprocessed dataset from affecting the model training, the dataset was randomly shuffled. This is because this step is important to prevent sampling bias, ensuring that all classes of data (normal and attack) are randomly distributed across the entire dataset, so that the subsequent split training and test sets are statistically representative samples of the original data distribution, thus ensuring the effectiveness and generalization ability of model training and evaluation.

Through these steps, the study constructs a high-quality, balanced, and suitable dataset for DL model learning and training, laying a solid foundation for the subsequent network attack classification task.

### Proposed model

**Fig. 2:**
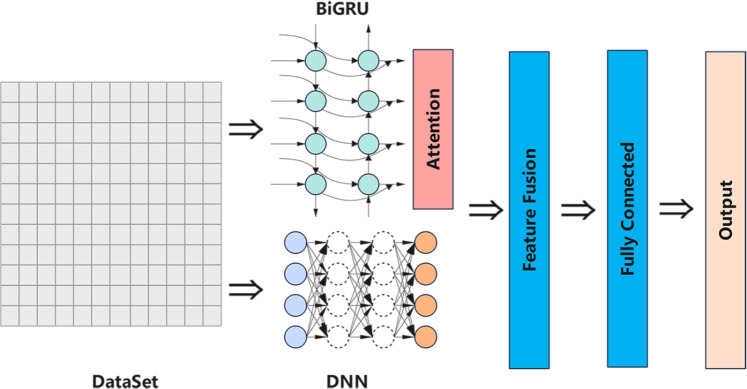
Multi-branch model architecture.

Our proposed model utilizes a multi-branch hybrid DL architecture as shown in Fig. 2 and Algorithm 1. This architecture combines the characteristics of DNN and BiGRU and enhances the global feature extraction ability of the model through the self-attention mechanism, efficiently extracts temporal and spatial characteristics from network traffic data, and finally improves detection accuracy. In addition, in order to improve performance further, a multi-branch feature fusion strategy and an improved training method are introduced. Specifically, first, the model works by receiving an input sequence with dimensions. Then, the proposed model processes the output in parallel:

1. In the first branch (DNN branch), the input sequence is flattened, and then this flattened vector is fed to the DNN module to extract global features.

2. In the second branch (BiGRU with Multi-Head Attention branch), the input sequence is fed to the BiGRU layer, which simultaneously outputs the hidden state sequence for all time steps and the final hidden state. Then, the model extracts the final forward hidden states and backward hidden states from the final hidden state and concatenates them to build a query vector. Finally, the query vector is fed into the Multi-Head Attention module together with the complete output sequence of BiGRU, so as to calculate the attention-weighted temporal features.

In the feature fusion stage, the global features from the DNN branch and the temporal features from the BiGRU-Attention branch are concatenated into a unified fused feature vector. Finally, the fusion feature vector is fed into the final classifier to generate the classification, which is then returned as the model’s output.

**Algorithm 1:**
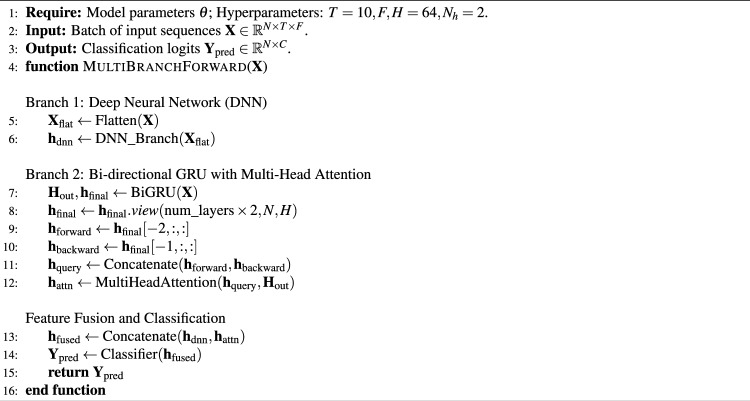
Multi-branch fusion model forward pass

DNN consists of two fully connected layers, the middle layer is batch normalization and ReLU activation function, and a Dropout layer is added after the ReLU activation. It mainly extracts high-level abstract features from the input data to improve the stability and nonlinearity of training and mitigate the risk of overfitting. Specifically, the time series data is first flattened, as shown in Equation (1), and then high-order features are extracted through two fully connected layers, as shown in Equations (2) and (3).

1 $$\begin{aligned} & {x_{flat}} = \mathrm{{Flatten}}(x) \in {R^{N \times (T \times F)}} \end{aligned}$$

2 $$\begin{aligned} & H^{(1)} = Dropout(ReLU(BN(x_{flat}W^{(1)}))) \end{aligned}$$

3 $$\begin{aligned} & H^{(2)} = Dropout(ReLU(BN(H^{(1)}W^{(2)}))) \end{aligned}$$

Here, $\mathrm{{N}}$ is the number of batch samples, $\mathrm{{T}}$ is the time step, and $\mathrm{{F}}$ is the feature dimension per unit time step, represents the flattening of the temporal dimension T and the feature dimension F into a single dimension, and are weight matrices, $\mathrm{{BN}}$ represents for batch normalization, and $\mathrm{{ReLU}}$ is the activation function.

**Fig. 3:**
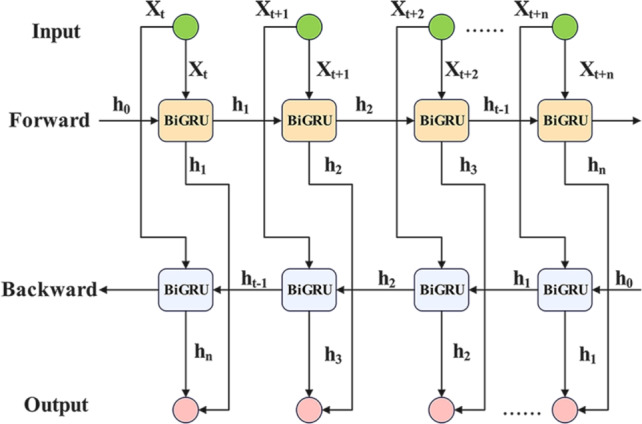
BiGRU structure.

BiGRU uses a single-layer Bidirectional GRU to process input sequence data forward and backward, capturing temporal dependencies in sequential network traffic data in an efficient manner. The BiGRU structure is shown in Fig. 3. The mathematical formula for calculating the forward and backward hidden states is as follows:

4 $$\begin{aligned} & {z_t} = \sigma ({W_z} \cdot [{h_{t - 1}},{x_t}]) \end{aligned}$$

5 $$\begin{aligned} & {r_t} = \sigma ({W_r} \cdot [{h_{t - 1}},{x_t}]) \end{aligned}$$

6 $$\begin{aligned} & {\tilde{h}_t} = \tanh ({W_h} \cdot [{r_t} \odot {h_{t - 1}},{x_t}]) \end{aligned}$$

7 $$\begin{aligned} & {h_t} = (1 - {z_t}) \odot {h_{t - 1}} + {z_t} \odot {\tilde{h}_t} \end{aligned}$$

Here, ${z_t}$ is the update gate, ${r_t}$ is the reset gate, ${h_t}$ is the candidate hidden state, ${\tilde{h}_t}$ is the final hidden state, $\sigma$ is the sigmoid activation function, and $\odot$ is the element-wise multiplication. Equation (8) illustrates the bidirectional temporal fusion process, where the final hidden state is obtained at each time step by concatenating the forward state and backward state. This dual-channel integration mechanism ensures comprehensive temporal feature capture, as follows:

8 $$\begin{aligned} h_t^{\mathrm{{bi}}} = [h_t^{\mathrm{{forward}}};h_t^{\mathrm{{backward}}}] \end{aligned}$$

The model’s capacity to focus on the most pertinent segments of the input sequence can be further improved by the attention mechanism. The output of the BiGRU layer is used as the input of the attention mechanism, which can selectively focus on different parts of the sequence and assign higher weights to features that are more indicative of specific attack types. The attention scores are calculated as follows:

9 $$\begin{aligned} \mathrm{{Attention}}(Q,K,V) = \mathrm{{softmax}}\left( {\frac{{Q{K^T}}}{{\sqrt{{d_k}} }}} \right) V \end{aligned}$$

The model employs a multi-head attention mechanism with num_heads=2, which enables the model to attend to different representation subspaces simultaneously. The multi-head attention is calculated as follows:

10 $$\begin{aligned} MultiHead(Q,K,V) = Concat(head_1, head_2, ..., head_h)W^O \end{aligned}$$

Here, $head_i = Attention(QW_i^Q, KW_i^K, VW_i^V)$, *Q* is the query matrix, *K* is the key matrix, *V* is the value matrix, ${d_k}$ is the dimension of each attention head, ${Q{K^T}}$ is the scaled dot-product, and *softmax* is the normalization function.

The attention mechanism takes two inputs: the query vector $h_combined$, which is the concatenation of the final forward and backward hidden states from BiGRU, and the key-value matrix consisting of all BiGRU output sequences. The query vector is computed as:

11 $$\begin{aligned} h_{combined} = [h_{final}^{forward}; h_{final}^{backward}] \end{aligned}$$

**Fig. 4:**
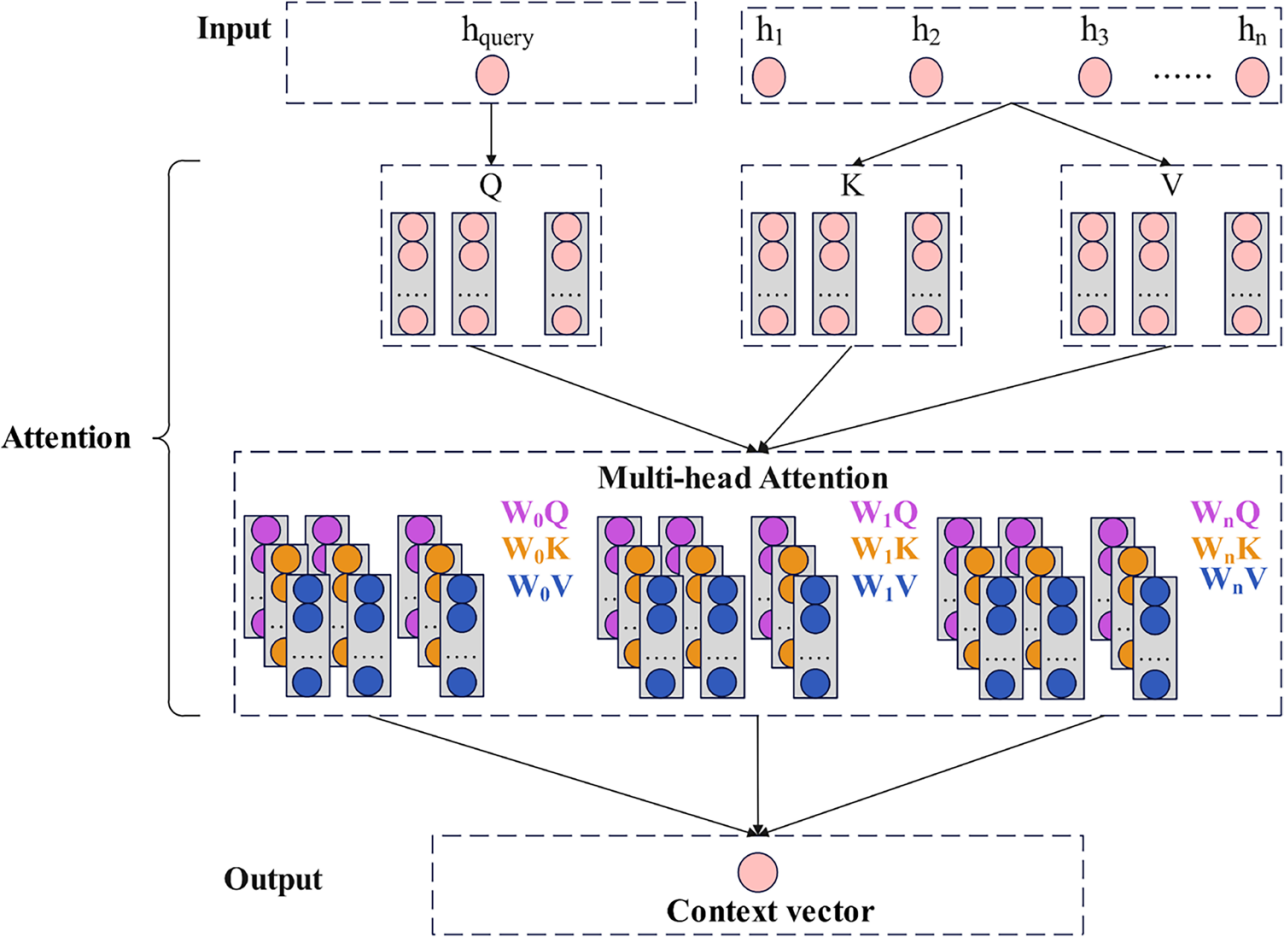
Multi-head attention structure.

The multi-head attention mechanism structure is shown in Fig. 4 and Algorithm 2.

**Algorithm 2:**
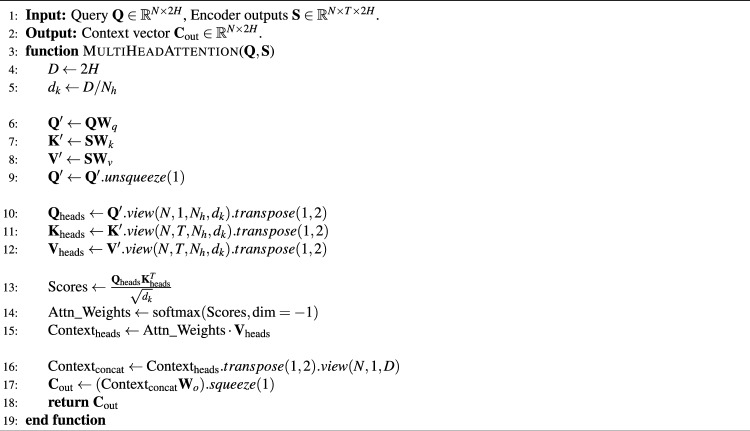
Multi-head attention mechanism

After obtaining features from both branches, the model performs feature fusion by concatenating the DNN output and the attention output:

12 $$\begin{aligned} h_{fused} = [h_{dnn}; h_{attn}] \end{aligned}$$

The fused features are then passed through a final classifier consisting of a fully connected layer with batch normalization, ReLU activation, and dropout for the final classification decision.

### Model design rationale

The proposed design is a multi-branch fusion architecture to extract multi-level features of time series data. Firstly, the DNN branch extracts the global characteristics of the data through the fully connected layer, which ensures that the model can generalize well in many kinds of network traffic. At the same time, the BiGRU branch establishes long-term temporal dependencies by utilizing the forward and backward GRU layers, which ensures that the model can capture context information and sequences that change over time. Secondly, the integration of multi-head attention enhances the ability of the model to focus on key time steps or key events within a sequence, which is crucial for identifying subtle anomalies or attacks. This fusion not only enhances the accuracy and robustness of the model but also provides scalability and generalization, enabling it to detect various types of cyber threats.

To achieve computational efficiency while maintaining performance, the model adopts several lightweight design strategies: utilizing a single-layer BiGRU (num_layers=1), reducing hidden dimensions (hidden_size=64), and implementing dual-head attention (num_heads=2). These design choices significantly reduce parameter count and computational overhead while mitigating overfitting risks. Table 2 shows the key parameters of the proposed model.

**Table 2:** Proposed model key parameters.

Architecture	Value	Training	Value
Sequence Length	10	Learning Rate	0.001
BiGRU Hidden Size	64	Batch Size	256
Attention Heads	2	Early Stopping	10
Dropout Rate	0.2	Optimizer	Adam

### Experimental design

This work was conducted on a laptop using NVIDIA CUDA acceleration technology, which can accelerate batch, parallel computation when training DL models and ensure that larger models complete training in a moderate amount of time. The experimental code was written in Python, and the PyTorch framework was used for model construction and training. Table 3 provides a summary of the experimental parameters used.

**Table 3:** Experimental parameters used.

Parameter	Value
CPU	Intel i7-12800HX
GPU	NVIDIA GeForce RTX4070 Laptop 8G
RAM	32GB
Operating System	Windows 11
Python	3.12.7
PyTorch	2.5.1+cu124
CUDA	12.6.65

This study mainly evaluates the performance of various DL models in 6G-IIoT threat detection tasks. To provide a fair and systematic evaluation of model performance, the experimental process and data partition strategy are as follows:

1. Data partitioning and training strategy: Using an 8:2 ratio to divide the training set and test set is a common approach in many studies on cyber threat detection^44–47^. Therefore, we create an $80\%$ training set and a $20\%$ test set from the preprocessed data set to ensure that the distribution of each class is similar in the training and testing phase, while reducing the impact of uneven data distribution on model performance.

2. Model training and optimization: In the training process, all models are trained for the same epoch, and a small batch size of 256 is used for random sampling. On the one hand, it can make full use of GPU hardware resources to improve the training speed, on the other hand, it provides a more stable gradient estimation than small batches, and avoids the convergence to “sharp” local minimum of too large batches^48^. Therefore, using a small batch size of 256 not only speeds up convergence, but also reduces the risk of overfitting.

At the same time, the optimizer used is Adam because it is very efficient in dealing with high-dimensional data and complex models^49^, and it is able to compute an adaptive learning rate for each parameter^50^. Although Adam’s default learning rate is usually 0.001^51^, we also set the initial learning rate to 0.001 to ensure stability for complex multi-branch hybrid models at the beginning of training, and then use the learning rate scheduler to dynamically adjust it.

### Evaluation metrics

To comprehensively evaluate each model’s actual performance in 6G-IIoT threat detection, various evaluation metrics are used, including accuracy, precision, recall, F1-score, and FPR.

Accuracy: The user measures the overall prediction accuracy and compares the performance of different models. The formula is shown as (13)^35^.

13 $$\begin{aligned} Acc = \frac{{T{P_{Attack}} + T{N_{Normal}}}}{{T{P_{Attack}} + T{N_{Normal}} + F{P_{Normal}} + F{N_{Attack}}}} \end{aligned}$$

Precision and Recall: These metrics show how well the model detects real attacks and reduces false positives in different attack datasets. Precision reflects the proportion of predicted attack samples that are really attacks, while Recall measures the percentage of real attacks that are successfully detected. The formula is shown as (14) and (15)^35^.

14 $$\begin{aligned} & Pr = \frac{{T{P_{Attack}}}}{{T{P_{Attack}} + F{P_{Normal}}}} \end{aligned}$$

15 $$\begin{aligned} & Rc = \frac{{T{P_{Attack}}}}{{T{P_{Attack}} + F{N_{Attack}}}} \end{aligned}$$

F1-score: Provides a more balanced evaluation in data distribution imbalanced scenarios, helping the model maintain robust performance under various attack types. The formula is shown as (16)^35^.

16 $$\begin{aligned} F1 = 2 \cdot \frac{{Precision \cdot Recall}}{{Precision + Recall}} \end{aligned}$$

FPR: In IIoT, false positive detection can have serious consequences such as business interruption and increased security costs. Calculating FPR helps understand the model’s ability to maintain high Recall while controlling the FPR. The formula is shown as (17)^52^.

17 $$\begin{aligned} \mathrm{{FPR}} = \frac{{\mathrm{{FP}}}}{{\mathrm{{FP}} + \mathrm{{TN}}}} \end{aligned}$$

To ensure the reliability and reproducibility of the experimental results, the data distribution and experimental environment were strictly controlled in the study design, and an independent training and testing process was used. First, the dataset was stratified by sampling, divided into train sets and test sets, to ensure that the various attacks were maintained in a similar distribution in the training and testing. The test set remained independent throughout the model training and adjustment process, did not participate in the optimization of model parameters and hyperparameters, and thus provided a real generalization performance measurement in the final evaluation stage. Secondly, all models were trained under the same number of training epochs, Batch Size, optimizer (Adam), and learning rate parameter to ensure that no external factors interfered with the comparison process. By consistently managing the input data shape and preprocessing steps, this study eliminated the potential bias between models caused by the input format of the data. At the same time, a fixed random seed is set in the code, and all model training and evaluation processes are conducted through a unified framework, making the evaluation results fairer and more reliable.

## Results and discussion

### Evaluation results

The proposed model shows a steady improvement trend in various metrics of the training set and the validation set, and the overall trend is quite close, and the difference in the final convergence point is not large, as shown in Table 4. The training accuracy increases from $94.63\%$ to $97.20\%$. The loss gradually decreased over the training process, while recall and F1 scores steadily increased to nearly $97\%$. The performance on the validation set also shows a similar upward trend; although the validation loss fluctuates slightly, the overall accuracy, recall, and F1-score are maintained above $96\%$. It shows that the model has a strong generalization capability.

**Table 4:** Training convergence summary.

Metric	Initial	Final	Improvement
Training Accuracy (%)	94.63	97.20	+2.57
Validation Accuracy (%)	96.13	96.88	+0.75
Training Loss	0.1275	0.0572	−55.14%
Validation Loss	0.0794	0.0691	−12.97%
Training FPR	0.0037	0.0019	−48.65%
Validation FPR	0.0026	0.0021	−19.23%

**Fig. 5:**
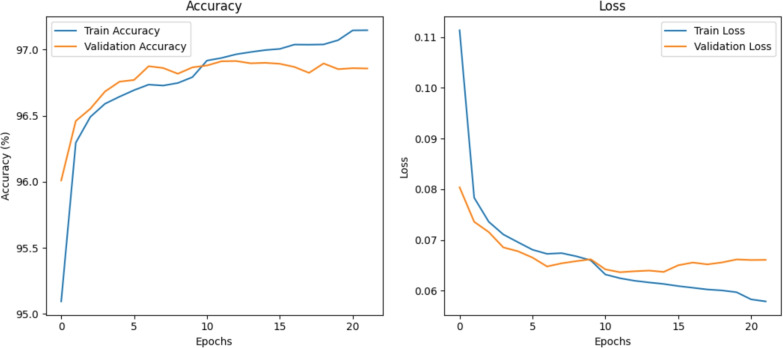
The accuracy and loss curves of the proposed model.

To prevent overfitting, by monitoring the loss curves of the model on the training and validation sets in Fig. 5, we observe that the training loss continuously decreases throughout the process, while the validation loss starts to increase after reaching its lowest point, and the training loss continues to decrease. This clearly indicates the occurrence of overfitting. To ensure the model’s best generalization ability, we implement an early stopping strategy; training is automatically stopped, and the final model is used to evaluate and report performance, effectively avoiding the impact of overfitting on model performance during subsequent training.

**Fig. 6:**
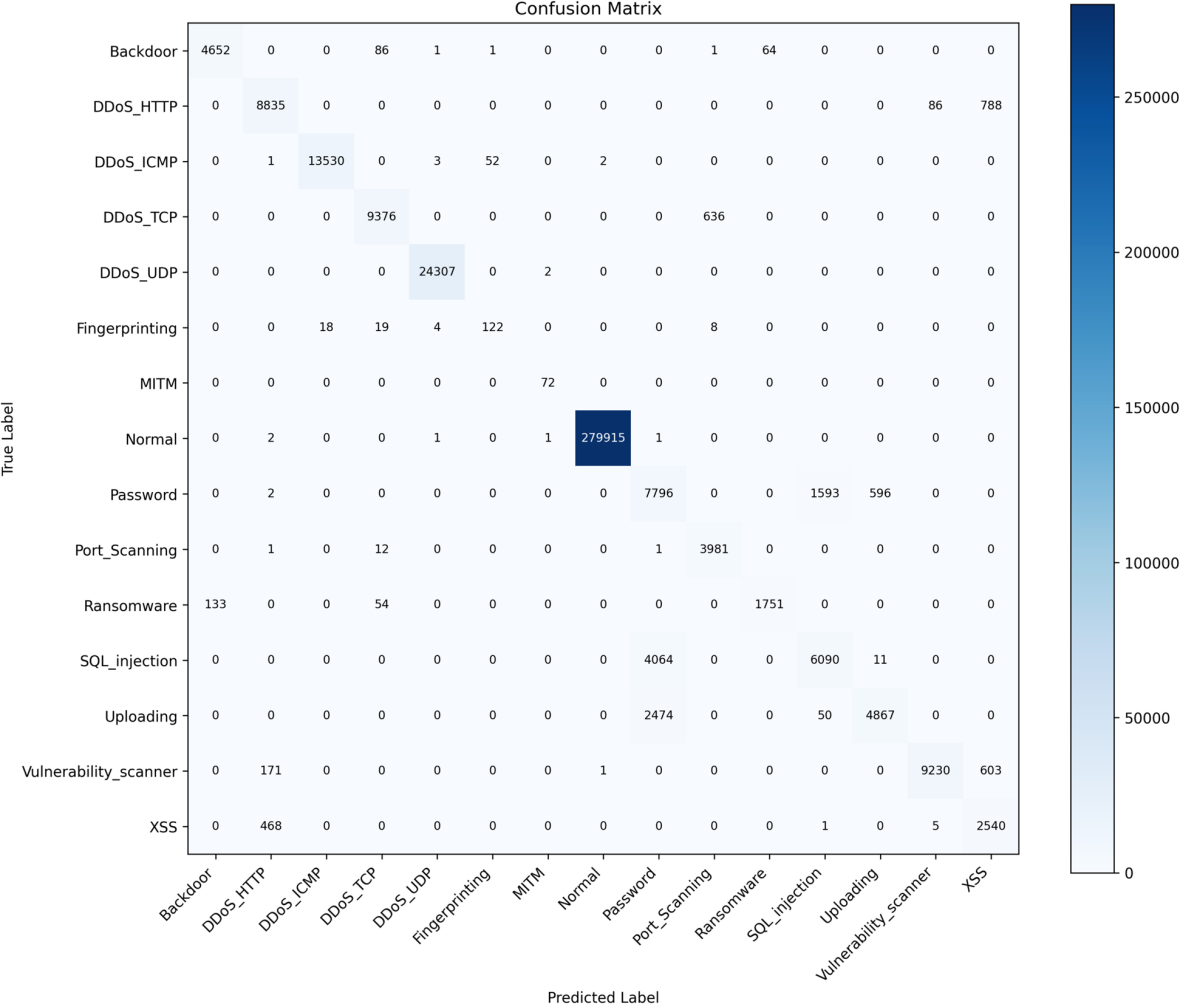
The confusion matrix of the proposed model.

The confusion matrix is used to compare the predicted value of a dataset with the actual value. It is a visual method to evaluate the performance of machine learning classification models. It is also suitable for DL in IIoT intrusion detection classification, so as to help us understand the model’s accuracy and effectiveness^53^. Figure 6 shows the confusion matrix of the Edge-IIoTset dataset, illustrating the classification results of the proposed model on this dataset. Firstly, there is perfect classification of the “Normal” category, but false positives also exist. However, false positives occur when distinguishing specific attack categories (such as port scanning, SQL injection), indicating that these different types of attacks may be very similar to initial stage network features in their early stages (such as request structure), thus resulting in insufficient discrimination. Overall, the model demonstrates stable detection capabilities and robustness on extremely imbalanced data.

**Fig. 7:**
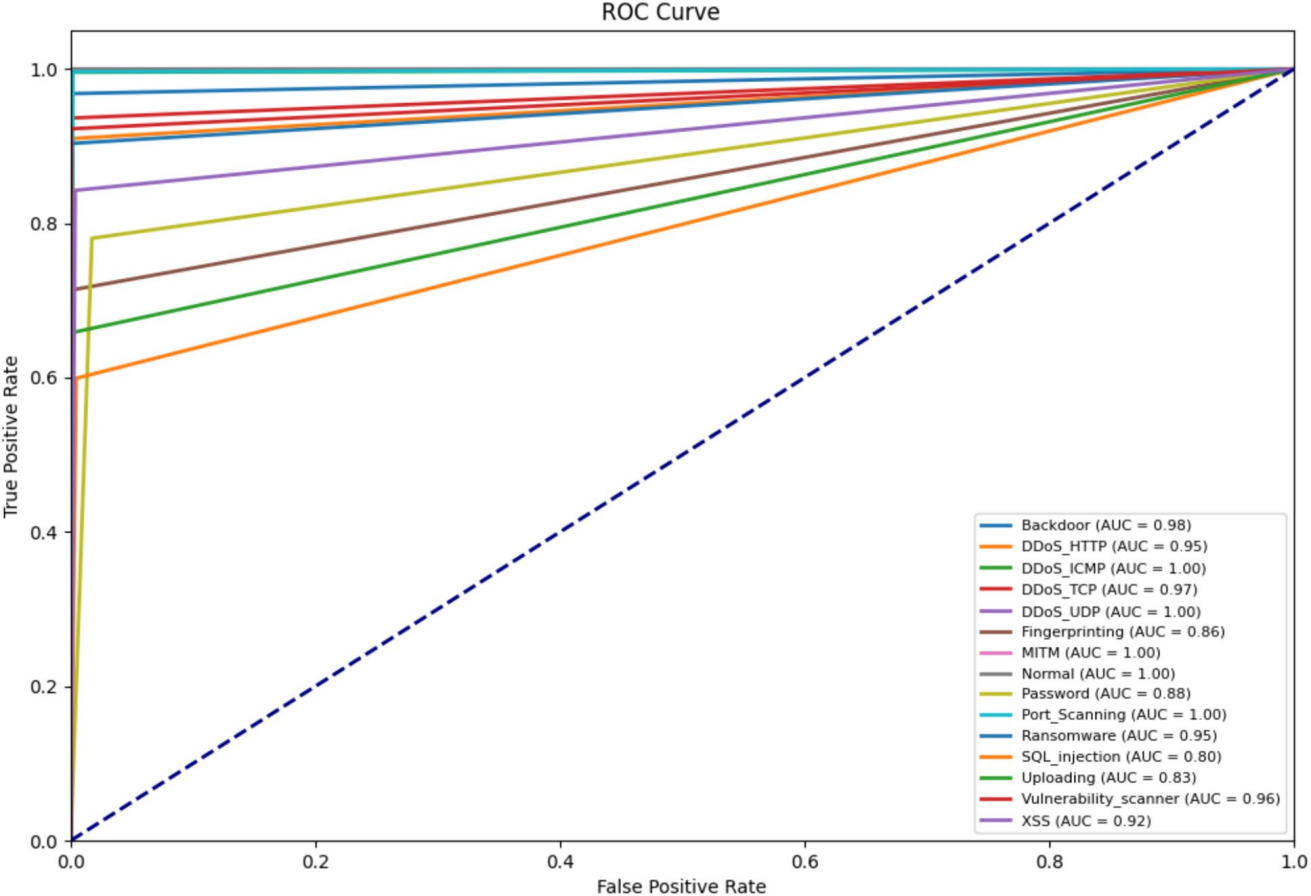
The ROC curves of the proposed model.

Figure 7 shows the Receiver Operating Characteristic (ROC) curves of the proposed model for all classes on the Edge-IIoTset dataset, along with the Area Under the Curve (AUC) value for each class. The ROC curves represent the trade-off between the True Positive Rate (TPR) and the FPR. It is clear from the figure that the curves for almost all classes are close to the top left corner of the figure, significantly higher than the diagonal dashed line representing random guessing (AUC=0.5), indicating that the model has excellent discrimination ability. In summary, the proposed model’s efficiency and robustness in multi-category classification tasks can maintain a high TPR while keeping the FPR at an extremely low level.

### Comparison with baseline and state-of-the-art

**Fig. 8:**
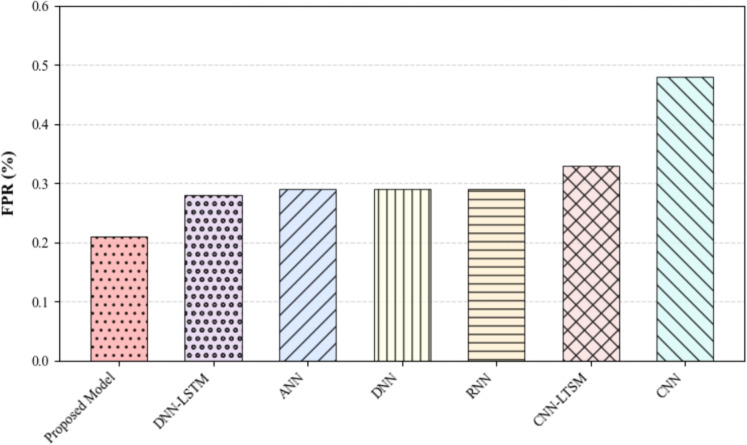
FPR of the proposed model and other models.

To compare the performance of the proposed model and the baseline models in the 6G-IIoT threat detection environment, this work uses a unified dataset and experimental process, and all models are trained and verified under the same conditions to ensure the fairness and comparability of the evaluation. The experimental results for all models are displayed in Table 5, and the Fig. 8 shows the FPR comparison between the proposed model and the baseline model.

The experimental results in the table show that the proposed DNN-BiGRU-Attention model outperforms the traditional baselines in 6G-IIoT threat detection, achieving significant performance improvement on all key metrics. By integrating the multi-branch structure, the model addresses the limitations of single-structure models, such as CNN and RNN, in recognizing complex threat patterns.

Table 5 shows that models such as DNN-LSTM, ANN, and DNN perform well in accuracy, precision, recall, and F1-score. However, the proposed model consistently performs better than any other model on these metrics. The evaluation results show that the accuracy reaches $96.88\%$, precision $97.23\%$, recall $96.88\%$, and F1-score $96.94\%$, which can capture the global dependence and time dependence in the data at the same time.

**Table 5:** Proposed model vs. Baseline model.

Model	Accuracy	Precision	Recall	F1-score	FPR
ANN	95.80%	96.03%	95.80%	95.83%	0.29%
CNN	93.07%	92.56%	93.07%	92.03%	0.48%
DNN	95.80%	95.86%	95.80%	95.79%	0.29%
RNN	95.79%	96.02%	95.79%	95.83%	0.29%
CNN-LSTM	95.19%	95.32%	95.19%	95.07%	0.33%
DNN-LSTM	95.85%	95.99%	95.85%	95.79%	0.28%
**DNN-BiGRU-Attention (Our Proposed Model)**	**96.88%**	**97.23%**	**96.88%**	**96.94%**	**0.21%**

Figure 8 shows the FPR of each model, where CNN shows the highest FPR, while the proposed model achieves the lowest FPR, demonstrating its superiority in minimizing false positives. Overall, the proposed model outperforms others and has superior performance across all key metrics.

To validate the effectiveness of our proposed model, we conducted a comprehensive comparison with several state-of-the-art related studies evaluated on the same Edge-IIoTset dataset^54–57^, as shown in Table 6. The results indicate that our proposed model demonstrates significant advantages in all key performance metrics, proving the effectiveness and excellence of our model architecture for intrusion detection in IIoT networks.

**Table 6:** Performance comparison between the proposed model and current research.

Work	Model	Dataset	Accuracy	Precision	Recall	F1-score
M. A. Ferrag et al.^35^	DNN	Edge-IIoTset	96.01%	$\times$	$\times$	$\times$
Rahamathulla et al.^54^	LSTM	Edge-IIoTset	93.87%	94.65%	91.47%	91.91%
Islam et al.^55^	BiLSTM	Edge-IIoTset	93.00%	93.86%	92.99%	92.87%
Yang et al.^56^	BiGRU-Attention-ICNN	Edge-IIoTset	94.70%	94.80%	94.70%	94.60%
Sasi et al.^57^	CNN-LSTM-ResNet-SA	Edge-IIoTset	33.30%	33.31%	100.00%	49.97%
**Our**	**DNN-BiGRU-Attention**	Edge-IIoTset	**96.88%**	**97.23%**	**96.88%**	**96.94%**

### Discussion

A benchmark comparison starting from ANN and DNN architectures is conducted to evaluate their ability to handle high-dimensional features. DNN has more hidden layers and neurons than ANN to capture more complex feature patterns. Then, CNN was introduced, treating features as one-dimensional data inputs to CNN, utilizing its convolutional kernels to capture local features, and using full pooling layers to achieve spatial compression, automatically learning local features at specific feature dimensions.

To model temporal dependence, RNNs (including LSTM) are used to process time-series features, capturing dynamic features of the traffic data as it evolves over time, helping to detect persistent or slowly evolving attacks.

To pursue higher prediction performance and robustness, these models are integrated as hybrid models that combine the strengths of different architectures to take advantage of complementary feature extraction and pattern recognition capabilities.

Finally, a model combining DNN, BiGRU, and Multi-head Attention was designed and achieves state-of-the-art performance. By capturing global features and temporal dependencies, and dynamically focusing on the most relevant features. The design allows the model adjust its dynamically focus on key features and time series, making it particularly suitable for the complex network environment of 6G-IIoT. The model can mine potential abnormal behavior from multi-dimensional traffic data and effectively identify persistent or periodic threats by taking advantage of the complementary advantages of multiple architectures. At the same time, we observe in Table 6 that our DNN-BiGRU-Attention achieves the highest Accuracy and F1 on Edge-IIoTset. This demonstrates that this complementary fusion improves category segregation, which is consistent with higher precision and recall.

Compared with the baseline DL model, the multi-head attention mechanism of the proposed model can provide displayed attention weights for each time series, enabling the model to directly visualize the time steps important in attack detection. However, this model still has a “black box” nature due to the complexity of the hybrid architecture, so further research is needed to integrate interpretability techniques into the detection process in a practical IIoT environment.

The novel hybrid model proposed in this study shows significant performance advantages following a comparative analysis of multiple baseline models. Although the traditional ANN model achieves $95.80\%$ accuracy and $0.29\%$ FPR through fully connected layers and nonlinear activation functions, its feature extraction capability is limited by the neural network structure. Although CNN is good at local feature extraction, overfitting issues result in a validation accuracy of only $93.07\%$ and an FPR as high as $0.48\%$. DNN reduces the verification loss to 0.0862 and achieves comparable performance with ANN by deepening the fully connected layer. RNN achieves $95.79\%$ accuracy by relying on temporal modeling ability, but there are still limitations in complex feature fusion. In the hybrid model, the serial structure of CNN-LSTM fails to achieve the expected performance superposition, and the verification accuracy is only $95.19\%$. Although DNN-LSTM controls the FPR at $0.28\%$, the overall improvement is limited, reaching $95.85\%$ accuracy. In contrast, the proposed model achieves enhanced performance through a multi-dimensional feature fusion mechanism in this work.

The evaluation results show that the model gradually improves with multiple training rounds. Through the early stopping mechanism, it monitors the validation loss and stops training when the improvement stabilizes. The model’s generalization ability to detect both positive and negative samples is demonstrated by the low FPR on both the training and validation sets. Overall, the model works well on the training and validation dataset without significant metric differences. This proves its reliability for practical applications needing accurate classification.

## Conclusion and future work

This paper proposes a hybrid model combining DNN, BiGRU, and an attention mechanism to effectively detect and classify cyber threats in 6G-IIoT networks. The multi-dimensional feature fusion architecture of the model combines the global feature extraction of DNN with the bidirectional temporal dependence capture of BiGRU, while the attention mechanism selectively focuses on key abnormal patterns. The proposed design enables robust processing of complex multidimensional network traffic data, effectively discovering potential abnormal behavior and persistent threats. Experimental results show that compared with the traditional baseline model and state-of-the-art studies model, the proposed model achieves $96.88\%$ accuracy,$97.23\%$ precision, $96.94\%$ F1-score, and $0.21\%$ FPR. In general, the proposed model provides an efficient and reliable solution for network security protection in the 6G-IIoT environment, which has important theoretical significance and practical application value.

In the future, the model parameter fine-tuning framework should be further explored to enable the model to perceive network topology changes and new attack features in real time, as well as adversarial sample detection technology to improve the robustness in adversarial attack scenarios. It is also necessary to further study the realization of low-latency reasoning and millisecond response among multiple nodes through collaborative optimization techniques such as Model Splitting and Knowledge Distillation in the distributed edge computing environment of 6G space-ground integrated network. At the same time, it explores the integration of interpretability techniques into the threat detection process in the practical IIoT environment.

In addition, the proposed model needs to be evaluated on other diverse or real-world IIoT datasets to verify its robustness further.

## Data Availability

The dataset used in this study, named Edge-IIoTset, is a public dataset that can be found on the Kaggle website: https://www.kaggle.com/datasets/mohamedamineferrag/edgeiiotset-cyber-security-dataset-of-iot-iiot.
